# Hidden in Plain Sight: High Tacrolimus Metabolism Doubles Kidney Transplant Failure and Drives Infection Related Mortality

**DOI:** 10.3389/ti.2025.15207

**Published:** 2025-11-03

**Authors:** Caner Süsal, Bernd Döhler, Erol Demir, Walaa Ibrahim, Medhat Askar

**Affiliations:** 1 Institute of Immunology, Heidelberg University Hospital, Heidelberg, Germany; 2 Transplant Immunology Research Center of Excellence TIREX, Koç University School of Medicine, Istanbul, Türkiye; 3 Division of Nephrology, Department of Internal Medicine, Yeditepe University School of Medicine, Istanbul, Türkiye; 4 Department of Internal Medicine and Nephrology, Faculty of Medicine, Assiut University, Assiut, Egypt; 5 Health Sector and Department of Immunology, College of Medicine, Qatar University, Doha, Qatar

**Keywords:** kidney transplant, tacrolimus concentration-to-dose ratio, allograft loss, mortality, infection

## Abstract

Low tacrolimus trough concentration-to-dose ratio (CDR) is recognized as an indicator of high tacrolimus metabolism. However, its impact on long-term transplant outcomes and potential for clinical intervention remains unclear. In the largest study to date, we analyzed the impact of a low CDR at post-transplant year 1 on graft loss and patient mortality in 21,865 kidney transplants. We also performed a longitudinal analysis of CDR dynamics and conducted a genetic correlation in a subset of 1,257 patients. Low CDR at year 1 was significantly associated with increased hazards of graft failure (HR up to 2.80) and infection-related mortality (HR = 1.63), even in patients with therapeutic trough levels and good graft function. In the longitudinal analysis, normalizing initially low CDR by year 2 significantly improves graft survival. Low CDR was identified in a substantial proportion of the cohort (25.2%). Black, female, and younger recipients (<50 years) had higher odds of having a low CDR. The CYP3A5*1A genotype was also strongly associated with low CDR (approximately 8-fold higher odds). Patients with a low tacrolimus CDR represent a large high-risk population. The normalization of tacrolimus CDR through co-medication with diltiazem and reductions in steroid dosing may improve graft survival. Our findings support personalized tacrolimus management based on metabolic profiling and genetic testing.

## Graphical Abstract

**Figure:**
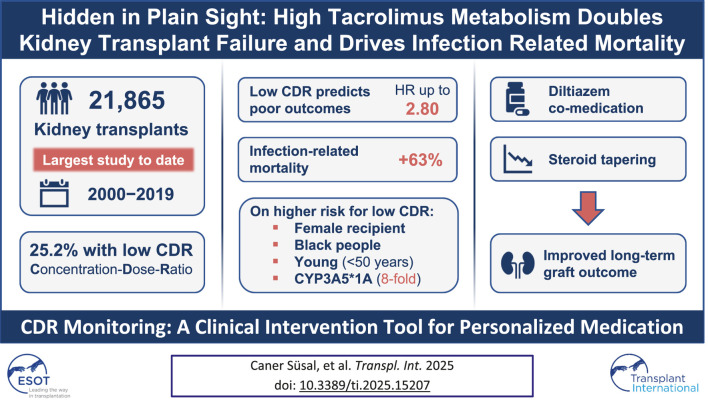

## Introduction

Tacrolimus as the most frequently used calcineurin inhibitor in kidney transplantation has a relatively narrow therapeutic range, yet despite decades of clinical use, a fundamental paradox persists in its therapeutic monitoring. While current clinical practice relies predominantly on blood trough concentration (C_0_) measurements to guide dosing decisions, mounting evidence suggests that this approach is not adequate to address the complex metabolic heterogeneity that profoundly influences transplant outcomes [1, 2]. A Collaborative Transplant Study (CTS) demonstrated that a trough level below 5 ng/mL at year 1 post-transplant is associated with significantly impaired graft survival [3]. However, rejection episodes and drug side effects occur also at trough levels 5 ng/mL or above [4].

The narrow therapeutic window of tacrolimus, combined with its significant intra- and inter-individual pharmacokinetic variability, calls for more sophisticated monitoring strategies that can identify high-risk patients before irreversible graft damage occurs. Therefore, besides C_0_ trough level, the ratio between the C_0_ concentration and dose (CDR) of tacrolimus as well as genetic variations in cytochrome enzymes involved in tacrolimus metabolism, have been investigated for their impact on kidney transplant outcomes [5, 6]. A low tacrolimus CDR was suggested as a modifiable risk factor to recognize high metabolizers who develop calcineurin inhibitor nephrotoxicity and BK nephropathy due to tacrolimus overdosing [7]. Others found that high metabolizers with a low CDR have an impaired kidney function due to acute rejection and delayed graft function caused by a too low tacrolimus trough level [8].

Variability in the genes of cytochrome P450 (CYP) 3A family of enzymes CYP3A4 and CYP3A5 has been shown to influence tacrolimus metabolism [9–11]. Expressors of the CYP3A5 enzyme homozygous or heterozygous for CYP3A5*1 allele have been reported to have increased tacrolimus clearance [12] and require higher doses to achieve the target tacrolimus concentration than non-expressors [13, 14]. A 1.5−2-fold higher starting dose of tacrolimus is recommended for CYP3A5 expressors [15]. Prevalence of patients expressing CYP3A5 is much higher among Black (40%–50%) and Asian (50%–70%) patients than among European ancestry patients (10%) [16, 17]. Carriers of certain *CYP3A4* alleles also exhibit varying levels of enzyme activity, including loss-of-function or low-function enzyme alleles [18, 19].

Overall, studies investigating the impact of tacrolimus metabolism on transplant outcomes and its association with genetic variations are confined to data generated from small-sized cohorts with short follow-up periods and were limited in representing different ancestries. We studied the impact of tacrolimus CDR on the robust endpoints “graft survival” and “patient mortality” in 21,865 kidney transplants, comprising 1,783 transplants in Black patients [20]. Additionally, we analyzed the dynamic impact of CDR changes over time and the factors associated with a high or low tacrolimus CDR.

## Methods

### Study Population and Data Collection

21,865 kidney transplantations performed during 2000–2019 in adult patients and reported to the CTS were investigated. Only patients with a functioning graft for more than 1 year, for whom 1-year tacrolimus dosage and C_0_ trough level data were available, were included; tacrolimus CDR was calculated from trough levels and doses at year 1, with additional year 2 data analyzed. Multi-organ transplantations (except simultaneous kidney-pancreas transplantation) and transplantations in patients receiving mammalian target of rapamycin-inhibitor (mTOR) or cyclosporine A in addition to tacrolimus were excluded.

### Exposures

CDR was calculated by dividing the measured trough concentration by the daily dosage. The impact of CDR on the three most common causes of death, namely infection (ICD-10 A00–B99), diseases of the circulatory system (I00–I99), and malignancies (C00–C97) was analyzed.

### Outcomes

Primary outcomes were the impact of the first-year CDR on all-cause graft failure, death-censored graft failure, and mortality during the second and third post-transplant years as well as the identification of factors associated with a low 1-year CDR of <1.05. The influence of CYP3A4 and CYP3A5 polymorphisms on death-censored graft failure was evaluated in a subgroup of 1,257 patients of European ancestry from Europe and North America with an available DNA sample.

### Statistical Analysis

CDR’s influence on outcomes was evaluated by Kaplan Meier estimates and Cox regression. Factors associated with low 1-year CDR were studied by logistic regression. For the analysis of the effect of tacrolimus CDR on the clinical outcomes, demographic, clinical, basic follow-up data, and transplant outcomes, including hospitalization for infection, graft failure, death-censored graft failure, and mortality were obtained from standardized follow-up questionnaires completed by the participating centers at 3-, 6-, and 12-month post-transplant and annually thereafter. An extended voluntary follow-up questionnaire with additional data, such as dose and trough level of immunosuppressive medication, was provided at years 1, 2, 3, and 5 post-transplant and every 5 years thereafter. The impact of the 1-year tacrolimus CDR (day*10^−3^/L) on outcomes during years 2 and 3 post-transplant as well as the combined influence of 1- and 2-year CDR on death-censored graft survival during years 3 and 4 post-transplant, were illustrated using Kaplan-Meier curves. All shown differences were confirmed through multivariable Cox regression analysis for a 2-year follow-up period to account for the potential influence of known factors on graft failure and patient mortality. The following confounders were included in all Cox regressions: geographical region, transplant year, first or retransplant, recipient and donor age, recipient sex and ancestry, original disease leading to end-stage renal failure, donor relationship (deceased, living), simultaneous kidney-pancreas transplantation, HLA-A+B+DR mismatches, time on dialysis prior to transplantation, general evaluation of the patient for transplant candidacy at the time of transplant, pretransplant antibodies, cause of donor death, marginal donor status, cold ischemia time (in the case of deceased donor), antibody induction, steroids, smoking, treatment for diabetes, and CDR at year 1. The use of antihypertensive drugs was recorded as a yes/no variable at the time of transplant admission. Other variables available at year 1, such as serum creatinine, tacrolimus trough level, and steroid dose could not be considered as confounders because of their significant correlation with CDR and were used only for stratification. A back-step elimination algorithm was used to exclude confounding factors with a threshold *P* value of >0.1. Interactions between the main exposure (CDR) and covariates pre-specified based on clinical relevance and prior literature (e.g., transplant number, recipient age, sex, ancestry, donor relationship, HLA mismatches, and original disease) were assessed. No interactions were statistically significant, and none were retained in the final models.

In the multivariable logistic regression analysis of factors associated with a low CDR <1.05 the following recipient-related pre-transplant variables were evaluated: transplant year, first or retransplant, recipient age, gender, and ancestry, treated for diabetes mellitus, original disease, smoking, pretransplant antibodies, preemptive transplant, medication (steroids, antibody induction, antihypertensive drugs), and in the genotyped subpopulation, in addition, the CYP3A5*1A and CYP3A4*22 genotypes. Quantitative data were presented as median and interquartile range, qualitative data as percentage and frequency. Chi-squared and Mann-Whitney U-tests were used for the comparison of the demographic characteristics. The software package IBM^®^ SPSS^®^ Statistics, version 29.0 (IBM Corporation, Armonk, NY, USA) was used. *P* values below 0.05 were considered statistically significant. Detail of genotypic analysis is described in Supplementary Material.

## Results

### Demographic and Clinical Characteristics

The demographics of the whole study cohort and the subgroup that was genotyped for *CYP3A4* and *CYP3A5* are shown in Table 1. 89.1% of all patients and 84.9% of the genotyped group were first graft recipients. As depicted in Supplementary Figure S1, genotyped patients demonstrated an almost identical all-cause graft survival as the subgroup of 9,349 patients of European ancestry who were not genotyped. Moreover, the 1-year tacrolimus trough and CDR levels were also not significantly different in genotyped versus not-genotyped recipients (*P* = 0.20 and *P* = 0.39, respectively). The vast majority of patients received immediate-release tacrolimus formulations, while a small proportion (6.6%) were on modified-release formulations (Advagraf^®^ or Envarsus^®^).

**TABLE 1:** Demographics of study patients (n, % or median with interquartile range).

Characteristic	All patients n = 21,865	Typing with CYP3A4, CYP3A5 n = 1,257
Events during post-tx years 2 and 3		
Graft failure	677	36
Death	729	33
Transplant year		
2000–2006	4,118 (18.8)	381 (30.3)
2007–2012	8,557 (39.1)	637 (50.7)
2013–2019	9,190 (42.0)	239 (19.0)
Geographical region		
Europe	10,059 (46.0)	806 (64.1)
Latin America	9,755 (44.6)	0 (0.0)
Other	2,051 (9.4)	451 (35.9)
Transplant number		
First transplant	19,480 (89.1)	1,067 (84.9)
Retransplant	2,385 (10.9)	190 (15.1)
Recipient sex		
Female	8,440 (38.6)	468 (37.2)
Male	13,425 (61.4)	789 (62.8)
Recipient age (years)	48 (37–58)	53 (42–62)
Recipient ethnic descent^a^		
European	15,562 (74.3)	1,257 (100.0)
Black African	1,783 (8.5)	0 (0.0)
Other	3,592 (17.2)	0 (0.0)
Donor relationship		
Deceased	15,446 (70.6)	1,090 (86.7)
Living	6,419 (29.4)	167 (13.3)
Donor age (years)	48 (37–57)	51 (40–59)
1-year serum creatinine (µmol/L)		
<130	12,829 (58.7)	752 (59.8)
≥130	9,036 (41.3)	505 (40.2)
1-year tacrolimus trough level (ng/mL)	7.3 (5.8–9.1)	7.3 (6.0–9.0)
1-year tacrolimus dose (mg/day)	4.0 (3.0–7.0)	4.0 (3.0–6.0)
1-year tacrolimus CDR (day*10^−3^/L)	1.60 (1.04–2.40)	1.69 (1.15–2.50)

Tx, transplant; CDR, trough level/dose ratio.

^a^ 4% unknown.

### Impact of CDR on the Outcomes

**FIGURE 1:**
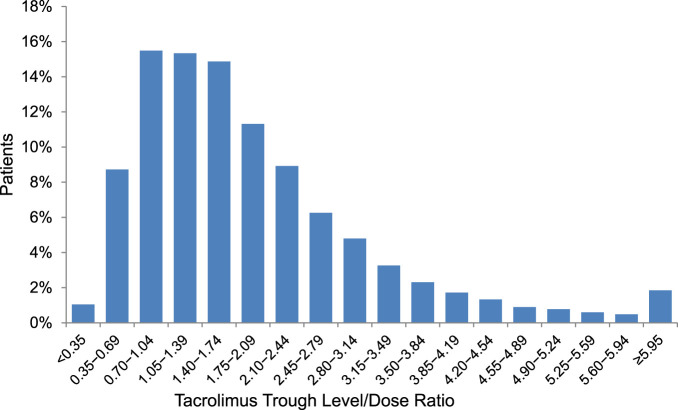
Distribution of tacrolimus trough level/dose ratio (day*10^−3^/L) at post-transplant year 1.

1-year CDR values of the cohort showed a right-skewed distribution making a subgrouping based on logarithmic values meaningful (Figure 1). Based on previously published studies [7, 21], we focused on lower CDR values, including 1.05, as they provided clinically meaningful insights and stratified the cohort into five groups using the CDR cut-offs 0.58, 0.78, 1.05, and 1.42, corresponding approximately to the equidistant ln(CDR) values −0.55, −0.25, 0.05, and 0.35.

**FIGURE 2:**
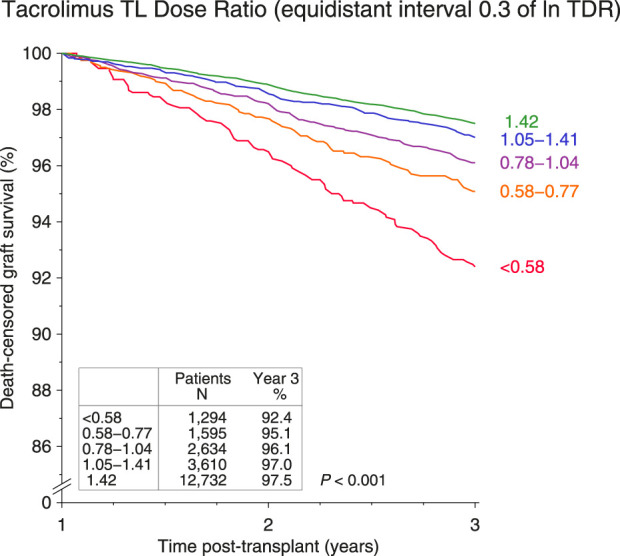
Influence of tacrolimus trough level/dose ratio (CDR, day*10^−3^/L) at year 1 on death-censored graft survival during post-transplant years 2 and 3 (log rank *P* value with trend of Kaplan-Meier analysis is shown).

In the Kaplan-Meier analysis, decreasing CDR values were associated with a stepwise decrease of death-censored graft survival (*P* < 0.001; Figure 2). An especially impaired graft survival was observed in patients with a very low 1-year CDR of <0.58, who made up 5.9% of the whole collective (n = 1,294).

In the multivariable Cox analysis, using CDR ≥1.42 as reference, CDR levels below 1.05 were associated with a significantly and progressively increased hazard of all-cause and death-censored graft failure, with the highest hazard observed in patients with very low CDR of <0.58 (all-cause failure, hazard ratio HR = 1.32 for CDR 0.78−<1.05, HR = 1.55 for CDR 0.58−<0.78, and HR = 2.05 for CDR <0.58; death-censored failure, HR = 1.47 for CDR 0.78−<1.05, HR = 1.69 for CDR 0.58−<0.78, and HR = 2.80 for CDR <0.58; *P* < 0.001 for all; Table 2). The impact of CDR on mortality was less pronounced and reached statistical significance starting at values below 0.78 (HR = 1.48 for CDR 0.58−<0.78 and HR = 1.44 for CDR <0.58; *P* = 0.003 and 0.019, respectively; Table 2).

**TABLE 2:** Influence of categorized trough level/dose ratio (CDR, day*10^−3^/L) at year 1 on all-cause graft failure, death-censored graft failure and patient mortality during second and third post-transplant years. Multivariable Cox regressions are used to calculate the hazard ratios (HR) with 95% confidence interval (CI).

CDR (day*10^−3^/L)	All-cause graft failure		Death-censored graft failure		Patient mortality	
	HR	95% CI *P* value	HR	95% CI *P* value	HR	95% CI *P* value
<0.58	**2.05**	1.70–2.47 <0.001	**2.80**	2.20–3.55 <0.001	**1.44**	1.06–1.96 0.019
0.58–<0.78	**1.55**	1.29–1.87 <0.001	**1.69**	1.31–2.19 <0.001	**1.48**	1.14–1.93 0.003
0.78–<1.05	**1.32**	1.12–1.55 0.001	**1.47**	1.17–1.86 <0.001	1.18	0.94–1.49 0.15
1.05–<1.42	1.05	0.90–1.23 0.54	1.17	0.93–1.46 0.18	0.98	0.79–1.22 0.87
≥1.42	1.00	Ref.	1.00	Ref.	1.00	Ref.

Overview of the covariates included and excluded from the final models is provided in Supplementary Table S2; univariable results are presented in Supplementary Table S3. Bold numbers indicate statistically significant HR values.

**FIGURE 3:**
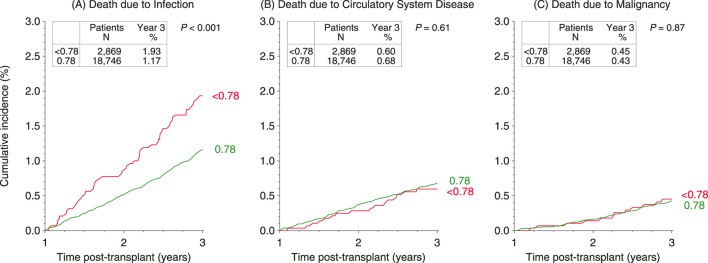
Influence of tacrolimus trough level/dose ratio (CDR, day*10^−3^/L) at year 1 on cumulative incidence of death due to **(A)** infection, **(B)** circulatory system disease, and **(C)** malignancy during post-transplant years 2 and 3 (log rank *P* values of Kaplan-Meier analyses are shown).

Patients with a low CDR of <0.78 and a functioning graft at year 2 were more frequently hospitalized for infection during year 2 than patients with a CDR of ≥0.78 (11.8% vs. 9.5%; *P* = 0.004; data not shown). In line with this finding and as illustrated in Figure 3, also the cumulative incidence of death due to infection during years 2 and 3 was significantly higher in patients with a CDR of <0.78 compared to patients with a CDR of ≥0.78 (1.93% vs. 1.17%, *P* < 0.001; HR = 1.63, 95% CI 1.20–2.22; *P* = 0.002). In contrast, the incidence of death due to circulatory system disease or malignancy was similar in patients with a CDR of <0.78 and ≥0.78.

Table 3 demonstrates the impact on death-censored graft failure of very low (<0.58) and intermediately low (0.58−<1.05) CDR values in different patient subgroups, as compared to the reference group with a normal CDR of ≥1.05. A CDR below 0.58 significantly increased the hazard of graft failure in all analyzed subgroups, irrespective of recipient sex and age, donor relationship, 1-year creatinine value, 1-year tacrolimus trough level, and 1-year steroid dose. The only exception was the subgroup of ≥60-year-old recipients in whom the 58% higher hazard did not reach statistical significance (*P* = 0.28). The highest hazard was observed in female recipients (HR = 3.59; *P* < 0.001), and in 18–49-year-old younger recipients, the hazard of graft failure increased approximately three-fold (HR = 2.99; *P* < 0.001). An intermediately low CDR of 0.58−<1.05 was also associated with a significantly increased hazard of graft failure in almost all subgroups, except for ≥60-year-old recipients, living donor recipients, and recipients with a 1-year tacrolimus trough level below 4.5 ng/mL or 1-year steroid dose of >5.0 mg/day. Even in patients with a 1-year tacrolimus trough level of ≥4.5 ng/mL, a very low CDR was associated with a 2.55-fold and an intermediately low CDR with a 1.51-fold increased hazard of failure (*P* < 0.001 for both).

**TABLE 3:** Influence of 1-year trough level/dose ratio (CDR, day*10^−3^/L) on death-censored graft failure during second and third post-transplant years in different subpopulations. Multivariable Cox regressions were used to calculate the hazard ratios (HR) with 95% confidence interval (CI) and CDR ≥1.05 as reference.

Subpopulation	N	CDR<0.58			CDR 0.58 – <1.05		
		HR	95% CI	P	HR	95% CI	P
All patients	21,865	**2.76**	2.20–3.47	<0.001	**1.52**	1.27–1.82	<0.001
Recipient sex							
Female	8,440	**3.59**	2.61–4.94	<0.001	**1.46**	1.10–1.92	0.008
Male	13,425	**2.15**	1.53–3.02	<0.001	**1.57**	1.24–1.99	<0.001
Recipient age							
18–49 years	11,849	**2.99**	2.30–3.91	<0.001	**1.58**	1.26–1.97	<0.001
50–59 years	5,421	**2.22**	1.23–3.99	0.008	**1.68**	1.13–2.48	0.010
≥60 years	4,595	1.58	0.69–3.62	0.28	1.11	0.66–1.86	0.70
Donor relationship							
Deceased	15,446	**2.87**	2.24–3.68	<0.001	**1.60**	1.31–1.94	<0.001
Living	6,419	**2.31**	1.31–4.08	0.004	1.19	0.74–1.92	0.47
1-year serum creatinine							
<130 μmol/L	12,829	**2.35**	1.39–3.99	0.001	**1.69**	1.16–2.47	0.006
≥130 μmol/L	9,036	**2.41**	1.87–3.11	<0.001	**1.33**	1.08–1.63	0.006
1-year trough level							
<4.5 ng/mL	1,888	**1.93**	1.23–3.04	0.005	1.10	0.70–1.75	0.68
≥4.5 ng/mL	19,977	**2.55**	1.92–3.39	<0.001	**1.51**	1.24–1.84	<0.001
1-year steroid dose							
≤5.0 mg/day	17,385	**2.73**	2.07–3.61	<0.001	**1.65**	1.34–2.03	<0.001
>5.0 mg/day	3,427	**2.20**	1.41–3.42	<0.001	1.16	0.79–1.71	0.45

Covariates: geographical region, donor relationship, transplant number, recipient and donor age, HLA mismatches, pretransplant antibodies, original disease, cause of donor death, marginal donor, smoking; univariable results are presented in Supplementary Table S4. Bold numbers indicate statistically significant HR values.

**FIGURE 4:**
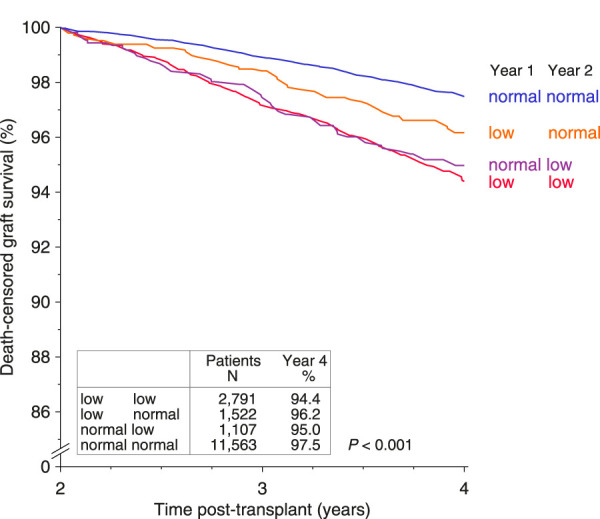
Combinatory analysis of the impact of low (<1.05) and normal (≥1.05) tacrolimus trough level/dose ratios (CDRs, day*10^−3^/L) at post-transplant years 1 and 2 on death-censored graft survival during years three and four post-transplant.

In a further subgroup analysis of 16,983 transplants with a follow-up of more than 2 years and an available CDR value also at year 2, we additionally analyzed the combinatory influence on death-censored graft survival of a low (<1.05) and normal (≥1.05) CDR at post-transplant years 1 and 2 by the Kaplan-Meier method (Figure 4). Irrespective of the level of CDR at year 1, an inferior graft survival was observed in patients with a low CDR at year 2, which was almost identical with that observed in patients with a low CDR at both post-transplant years. Approximately one-third of patients with a low 1-year CDR (35.3%) had a normal CDR at year 2. Importantly, compared to patients with a low CDR at both post-transplant years, a significantly better graft survival was observed in these patients (*P* = 0.013).

### Factors With an Influence on CDR Levels

**FIGURE 5:**
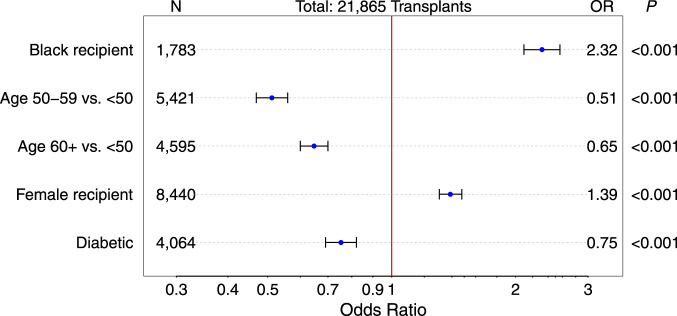
Odds ratios (OR) with 95% confidence interval of variables with significant influence on a low 1-year CDR below 1.05 days*10^−3^/L in multivariable logistic regression analysis (exact 95%-confidence intervals and results of univariable logistic regressions are presented in Supplementary Table S5).

As depicted in Figure 5, Black and female recipients showed a higher odds of having a low CDR below 1.05 at post-transplant year 1, whereas higher recipient age and treatment for diabetes were associated with a lower odds of a low 1-year CDR. Patients who were on diltiazem at year 1 showed a significantly lower incidence of a low 1-year CDR of <1.05 compared to patients not receiving diltiazem (15.5% vs. 25.7%; *P* < 0.001; Supplementary Figure S2). Moreover, a significant association was observed with steroid dosage: patients on a higher 1-year steroid dose (>5 mg/day) had worse CDR values (Supplementary Figure S3, *P* < 0.001). This observation corresponds with the results presented in Figure 4 when steroid dosages at 1 and 2 years post-transplant are taken into account. While the *low/low* and *normal/normal* groups showed average reductions in steroid dose during the second post-transplant year (−6.8% and −5.6%, respectively), the *normal/low* group, which experienced worsening CDR values, demonstrated a slight increase in steroid dose (+1.0%). In contrast, the *low/normal* group, which showed improved CDR values, exhibited the largest reduction in steroid dose during the second year (−9.7%). Consistent with these findings, renal outcomes also differed across groups. At year 3, median eGFR had declined in the *low/low* group (53 → 50 mL/min/1.73 m^2^), the *normal/normal* group (57 → 56 mL/min/1.73 m^2^), and the *normal/low* group (57 → 54 mL/min/1.73 m^2^). In contrast, the *low/normal* group maintained stable renal function, with mean eGFR values of 58 mL/min/1.73 m^2^ at both years 2 and 3.

**FIGURE 6:**
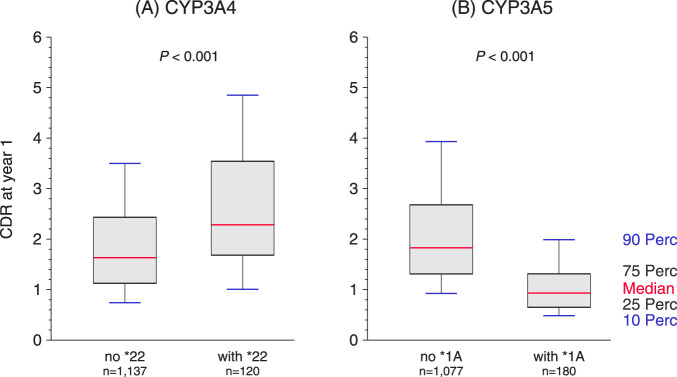
Association of tacrolimus concentration/dose ratio (CDR) at post-transplant year 1 with genetic polymorphisms in **(A)** CYP3A4 and **(B)** CYP3A5. Each panel illustrates the CDR distribution according to genotype to show the individual effects of each variant.

To analyze whether a low CDR at year 1 has a genetic background, a subgroup of 1,257 patients were typed for the *CYP3A4* and *CYP3A5* genes. Expressor genotypes of the CYP3A4 enzyme *CYP3A4* without *22 and of the CYP3A5 enzyme *CYP3A5* with *1A were present in 90.4% and 14.6% of patients, respectively; in both cases without a statistically significant deviation from the Hardy-Weinberg equilibrium (CYP3A4, *P* = 0.092; CYP3A5, *P* = 0.67; Supplementary Table S1). CYP3A5*1A expressor genotypes were significantly associated with a low CDR of <1.05 at year 1 (phi coefficient ϕ = 0.42, *P* < 0.001). In contrast, the association of the CYP3A4 expressor genotypes (*CYP3A4* without *22) with low CDR was statistically significant but much weaker (ϕ = 0.08, *P* = 0.005). Importantly, while the 1-year CDR values were significantly lower in patients carrying the CYP3A4 and CYP3A5 expressor genotypes than in patients without these genotypes (Figure 6; *P* < 0.001 for both), tacrolimus trough levels at year 1 did not differ significantly between patients with and without the expressor genotypes of these enzymes (CYP3A4: 7.20 [6.00–9.00] vs. 7.45 [6.05–9.00] ng/mL, *P* = 0.59: CYP3A5: 7.20 [6.00–9.10] vs. 7.40 [5.90–9.00] ng/mL, *P* = 0.84). In the multivariable logistic regression, including the same covariates, that were significant in the overall cohort, the CYP3A5*1A genotype was associated with an 8.1-fold higher odds of having a low CDR of <1.05 (95% CI: 4.8–13.6, *P* < 0.001), whereas CYP3A4*22 was not significantly associated with low CDR (*P* = 0.44, Supplementary Table S5). Presumably due to the low number of patients with the CYP3A5*1A genotypes (n = 180) and a low number of patients with death-censored graft failure in the typed subgroup (n = 36), the influence of the CYP3A5*1A genotype on death-censored graft failure did not reach statistical significance (HR = 1.60, 95% CI 0.72–3.53, *P* = 0.25).

## Discussion

In this largest study conducted so far to analyze the association between tacrolimus CDR and kidney transplant outcomes, we found a significant association between low CDR values at post-transplant year 1 and graft as well as patient survival during years 2 and 3. The hazard of all-cause and death-censored graft failure increased gradually at CDR values below 1.05, with a more than two-fold increased hazard at a very low CDR of <0.58. A CDR value below 1.05 was found in as many as 25.2% of patients with a functioning graft at year 1. Analysis of longitudinal CDR changes revealed that normalization of low CDR from year 1 to year 2 improved graft survival significantly, highlighting the potential for continuous CDR monitoring as a practical tool to optimize immunosuppression. Efforts to normalize CDR by addition of diltiazem and reduction of steroid dosing might be beneficial even in patients with normal serum creatinine (<130 μmol/L), corresponding to eGFR >60 mL/min/1.73 m^2^. Low CDR was also associated with increased patient mortality; however, the effect was slightly less pronounced with increasingly significant association at values below 0.78. Importantly, the strong association between low CDR and higher death-censored graft failure was independent of recipient sex, recipient age, and donor relationship and was present also in patients with good graft function and a normal 1-year tacrolimus trough level.

As in our study, high tacrolimus metabolizers were defined as individuals with a low CDR of <1.05 in three other studies [7, 21, 22]. These findings, along with the incremental impact of post-transplant CDR on graft and patient survival and its strong effect in all important patient subpopulations, highlight the strength of this parameter in predicting kidney transplant outcomes.

Prior studies investigating the association between *CYP3A5* genotypes and clinical outcomes showed conflicting results. While some studies reported no association between genotypes and clinical outcomes [23, 24], others reported an association between expressors and poor clinical outcomes, including significantly higher incidence of delayed graft function and biopsy-proven rejections [25] and higher death-censored and all cause graft failure in Black kidney transplant recipients [26] and significantly higher frequency of *de novo* donor-specific HLA antibody development and antibody-mediated rejection [27]. Therefore, the Clinical Pharmacogenetics Implementation Consortium Guidelines made a strong recommendation to use a higher starting dose than the standard tacrolimus dose in CYP3A5 expressors [15]. On the other hand, this guideline concluded that more data are needed to understand whether genotype-based dosing will affect the clinical outcomes. Our study provides a critical element of the missing data by reporting a significant association between a low CDR and increased patient mortality, which was particularly attributable to deaths from infection. Also, the absolute risk difference for hospitalization due to infection during the second post-transplant year was 2.3% indicating the clinical relevance of the severity of infections in kidney transplant recipients. This finding suggests that patients with low CDR, potentially due to higher exposure to tacrolimus metabolites, face increased morbidity, supporting the need for CDR normalization to mitigate these outcomes. Despite having a normal tacrolimus trough level, patients with a low CDR are at an increased hazard of infection-related death due to increased tacrolimus exposure. However, not unexpectedly, an association of low CDR with mortality due to malignancy or cardiovascular events during years 2 and 3 post-transplant could not be demonstrated as tacrolimus use is associated mainly with an increased incidence of tumors with lower mortality rates, such as skin cancer and Kaposi’s sarcoma [28], and tacrolimus-related metabolic complications, such as hypertension, dyslipidemia, and diabetes mellitus, increase mortality in later phases after transplantation [29]. Schütte-Nütgen et al. observed a similar, but not significant trend between a low CDR below 1.05 and patient mortality [21]. In contrast, Bartlett et al. found a higher mortality rate in patients with a high CDR, most probably due to the 2.04 CDR cut-off used in this study being too high [30]. In our study, CDR values significantly associated with mortality were with <0.78 much lower.

In line with previous studies, also in our large cohort, Black and female recipients had higher odds of having a low 1-year CDR (Figure 5), most probably due to the previously reported higher frequency of certain cytochrome enzyme genes in Black patients and increased cytochrome enzyme activity in females [31–33]. These findings suggest potential disparities in CDR levels related to ancestry and sex, underscoring the need for tailored approaches to improve equity in transplant care. An interaction between diltiazem, a CYP450 inhibitor, and tacrolimus has also been reported [34, 35], Our finding on the reduced incidence of low CDR in patients on diltiazem is consistent with its CYP450 inhibitory properties that can lead to decreased tacrolimus clearance and suggests inclusion of diltiazem to the patient’s drug protocol as a potential therapeutic strategy to mitigate high metabolism. No significant interaction was observed between CDR values and transplant number.

In a subgroup analysis of 1,257 patients, we obtained evidence that CYP3A5*1A genotypes are associated with higher odds of having a low CDR, whereas the expressor genotypes of the other cytochrome enzyme CYP3A4, namely *CYP3A4* without *22, were only weakly associated with low CDR. *CYP3A5* genotyping has proven effective in guiding tacrolimus dosing with CYP3A5 expressors requiring approximately 50% higher doses to reach the target therapeutic range compared to non-expressors [36]. These findings altogether indicate that recipients testing for the CYP3A5*1A polymorphism can identify patients at increased risk of having low CDR and it can be hypothesized that these patients would benefit from steroid dose tapering and the addition of diltiazem into their treatment regimen.

Conversely, the significantly lower odds of elderly patients having a low CDR at year 1 could be due to a decrease in the activity of enzymes involved in the drug’s distribution, metabolism, excretion, and clearance at higher age [37]. The association of diabetes mellitus with low CDR at year 1 could be explained by the gastric delay associated with diabetic neuropathy [38, 39].

Although the 1-year CDR values were significantly lower in patients carrying the CYP3A4 and CYP3A5 expressor genotypes than patients without these genotypes, tacrolimus trough levels at year 1 did not differ significantly between patients with and without the expressor genotypes, indicating that the adjustment of tacrolimus dose to its trough level is an effectively practiced standard of care. Despite the strong impact of low CDR on the outcomes and despite the strong association of the CYP3A5*1A genotypes with low CDR in our study and a previous meta-analysis [40], the influence of this genotype on death-censored graft failure did not reach statistical significance, most probably due to the low percentage of patients with this genotype and low number of patients with graft loss in our genotyped subgroup.

The main strength of our study is the high number of recipients of deceased as well living donor kidney transplants from different ancestry backgrounds, across 129 different transplant centers in 31 countries, which minimizes bias from single-center practices, and provides a robust foundation for a statistically validated analysis of the CDR effect on the hard outcome measures “graft loss” and “patient mortality.” While variability in co-medications could influence CDR, our multivariable regression analyses adjusted for co-medications and additional subgroup analyses in patients with a high and low steroid dose confirm the consistent association of low CDR with adverse outcomes. As a retrospective registry analysis, however, we could only identify associations but not causality because biopsy results, that would allow an analysis of the specific causes of allograft failure, were not captured in CTS due to high variation of results reported by different pathologists [41]. Therefore, future randomized controlled trials are warranted to confirm the association between tacrolimus CDR normalization and improved graft and patient survival, as well as to assess the contribution of tacrolimus metabolites to toxicity in patients with low CDR. However, low CDR was linked with BK virus infection and CNI toxicity in previous studies [7] so that a causal contribution of these factors to increased graft loss rates observed in our patients with low CDR is biologically plausible. CDR values prior to year 1 are not available in CTS which means that any clinically significant variation of CDR level during the first year was not captured. However, in all important patient subgroups, a progressively increasing hazard of graft failure was observed with gradually decreasing CDR values, underlining the robustness of this parameter in predicting the clinical outcomes. Moreover, the analyzed 1- and 2-year values can be considered as the best choice because they are obtained in a stable phase of medication after transplantation. Lastly, analyses were not adjusted for post-transplant donor-specific HLA antibodies (DSA), as routine DSA monitoring was not yet standard in the majority of transplant centers during the study period. Pretransplant panel reactive HLA antibodies and HLA mismatches were included, which allowed partial coverage of alloantibody effects.

The combined analysis of the impact of 1- and 2-year CDR values on death-censored graft survival revealed the important finding that improvement of a low 1-year CDR to a normal value at year 2 results in improved graft survival, highlighting the importance of continuous monitoring and actively normalizing CDR values throughout the entire post-transplant period. Our finding that approximately two-third of patients with a low 1-year CDR still had a low CDR at year 2 indicates the substantial potential for improving the clinical outcomes in kidney transplantation by considering CDR. This stratification by CDR trajectories further underscored the clinical relevance of CDR normalization. Patients with persistently low CDR values (low/low), persistently normal values (normal/normal), or declining values (normal/low) all showed a decrease in median eGFR between year 2 and year 3 (e.g., 53 → 50, 57 → 56, and 57 → 54 mL/min/1.73 m^2^, respectively). By contrast, patients who shifted from low to normal CDR values (low/normal) maintained stable renal function, with mean eGFR remaining unchanged at 52 mL/min/1.73 m^2^. These findings suggest that normalization of initially low CDR values is not associated with nephrotoxicity but may instead contribute to preservation of renal function.

In our cohort, changes in measured CDR over time were observed and appeared to correspond with modifiable clinical factors. Specifically, co-medication with diltiazem and reductions in steroid dosing were associated with higher CDR values, whereas patients with increasing steroid doses showed declining CDR values (Figure 4; Supplementary Figures S2–S3). Thus, while the underlying CYP3A genotype is fixed, the measured CDR captures both genetic and modifiable influences, and its normalization can reflect clinically actionable changes that are associated with improved graft survival.

## Conclusion

Findings of our large-scale study strongly indicate that monitoring of tacrolimus CDR, as a simple and cost-effective tool, can assist physicians in their daily clinical routine to identify tacrolimus-treated kidney transplant recipients at risk of inferior outcomes, even if they have good graft function and their tacrolimus trough levels are within the therapeutic range. Genetic analysis of cytochrome enzymes could be useful in patients with a low CDR to find out whether the reason for the increased tacrolimus metabolism has a genetic background.

## Data Availability

The data analyzed in this study is subject to the following licenses/restrictions: The raw data are available upon request to the Collaborative Transplant Study in accordance with the consents of the patients and the participating transplant centers and registries. Requests to access these datasets should be directed to csusal@ku.edu.tr.
