# Corrigendum: Hidden in Plain Sight: High Tacrolimus Metabolism Doubles Kidney Transplant Failure and Drives Infection Related Mortality

**DOI:** 10.3389/ti.2025.15860

**Published:** 2025-12-17

**Authors:** Caner Süsal, Bernd Döhler, Erol Demir, Walaa Ibrahim, Medhat Askar

**Affiliations:** 1 Institute of Immunology, Heidelberg University Hospital, Heidelberg, Germany; 2 Transplant Immunology Research Center of Excellence TIREX, Koç University School of Medicine, Istanbul, Türkiye; 3 Division of Nephrology, Department of Internal Medicine, Yeditepe University School of Medicine, Istanbul, Türkiye; 4 Department of Internal Medicine and Nephrology, Faculty of Medicine, Assiut University, Assiut, Egypt; 5 Health Sector and Department of Immunology, College of Medicine, Qatar University, Doha, Qatar

**Keywords:** kidney transplant, tacrolimus concentration-to-dose ratio, allograft loss, mortality, infection

## Abstract

**Figure:**
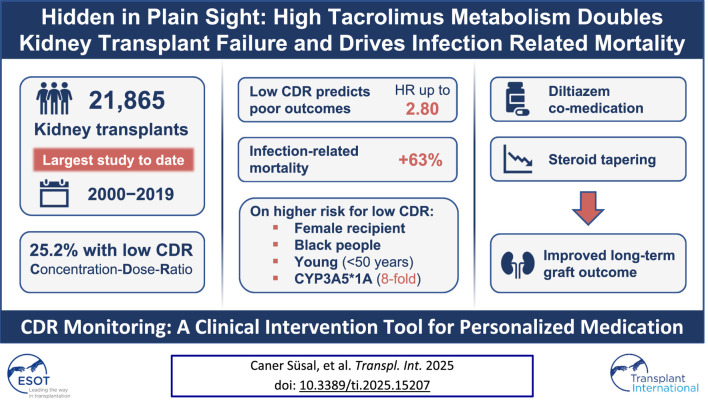

In the published article, there was an error in the article title. Instead of “*Hidden in Plain Sight: Low Tacrolimus Metabolism Doubles Kidney Transplant Failure and Drives Infection Related Mortality*”, it should be “*Hidden in Plain Sight: High Tacrolimus Metabolism Doubles Kidney Transplant Failure and Drives Infection Related Mortality*”.

The authors apologize for this error and state that this does not change the scientific conclusions of the article in any way. The original article has been updated.

## Generative AI statement

Any alternative text (alt text) provided alongside figures in this article has been generated by Frontiers with the support of artificial intelligence and reasonable efforts have been made to ensure accuracy, including review by the authors wherever possible. If you identify any issues, please contact us.

