# Supplementary material for: Hidden in Plain Sight: High Tacrolimus Metabolism Doubles Kidney Transplant Failure and Drives Infection Related Mortality
Source: Transpl Int. 2025 Nov 3;38:15207. doi: 10.3389/ti.2025.15207 (PMC12620305; doi:10.3389/ti.2025.15207)

Supplementary Material

Methods for Genotype

For *CYP3A4* and *CYP3A5* genotyping, the commercially available MassArray-based VeriDose® Core Panel reagents and the MassARRAY® System instrument were utilized (Agena Bioscience, San Diego, CA). Genotypes and * allele designations were assigned using the Translational Software commercial bioinformatics pipeline (Mercer Island, WA). *CYP3A4* and *CYP3A5* polymorphisms were determined using DNA samples from the CTS biobank, with selection based merely on availability of DNA. Genotyping was performed to assess the functional status of the *CYP3A5* gene, focusing on two key single nucleotide polymorphisms (SNPs): *rs776746 (*3, c.6986A>G) and rs10264272 (*6, c.14690G>A)*. These SNPs correspond to loss-of-function alleles, with **3* and **6* leading to reduced or absent CYP3A5 enzyme activity, respectively, in contrast to the functional (wild-type) **1A* allele. DNA samples were analyzed to determine only the presence of **1, *3, and *6* alleles. Individuals were classified based on the presence or absence of at least one functional **1* allele. Patients carrying at least one **1* allele *(*1/*1, *1/*3, or *1/*6)* were categorized as expressors of the CYP3A5 enzyme whereas those homozygous for the loss-of-function alleles **3/*3, *3/*6, or *6/*6* were classified as non-expressors. This binary classification was adopted due to the low frequency of **1/*1* homozygotes (n=5) in the study cohort, which limited the statistical power for further stratification into normal, intermediate, and poor metabolizers. Also, patients with *CYP3A4*22* genotype were classified as low-expressors and patients with non-*22 *CYP3A4* alleles as expressors of the CYP3A4 enzyme.

Table S1. Distribution of genotypes with *P* value of Hardy-Weinberg equilibrium (chi-square test).

Genotype	Observed	Percent	Expected	*P*
CYP3A4				0.67
*1/*1	1,124	89.4%	1,126	
*1/*2	13	1.0%	13	
*1/*22	118	9.4%	115	
*2/*2	0	0.0%	0	
*2/*22	1	0.1%	1	
*22/*22	1	0.1%	3	
CYP3A5				0.092
*1A/*1A	5	0.4%	7	
*1A/*3	173	13.8%	171	
*1A/*6	2	0.2%	0	
*3/*3	1,073	85.4%	1,073	
*3/*6	4	0.3%	6	
*6/*6	0	0.0%	0	

Table S2. Overview of the final models for the multivariable Cox regression results presented in Table 2 which shows the influence of categorized trough level/dose ratio (CDR, day*10–3/L) at year 1 on all-cause graft failure, death-censored graft failure and patient mortality during second and third post-transplant years. Covariates included in the model are marked with "x," while excluded covariates are indicated with "–".

Covariates	All cause GF	Death-censored GF	Mortality
Geographical region	x	x	x
Tx year	–	–	x
Tx number	x	x	x
Recipient age	x	x	x
Donor age	x	x	x
Recipient sex	–	–	–
Recipient ancestry	x	x	–
Original disease	x	x	x
Donor relationship	x	x	x
Kidney+pancreas tx	x	–	x
HLA-mismatches	x	x	–
Time on dialysis	–	–	–
General evaluation	x	–	x
Pre-Tx antibodies	x	x	–
Cause of donor death	x	x	–
Marginal donor	x	–	–
Cold ischemia time	–	–	–
Antibody induction	x	–	–
Steroids	x	x	–
Smoking	x	x	–
Treatment for diabetes	–	–	–
Antihypertensive drugs	x	–	x

Tx, transplant

Table S3. Influence of categorized trough level/dose ratio (CDR, day*10^{-3}/L) at year 1 on all-cause graft failure, death-censored graft failure and patient mortality during second and third post-transplant years. Univariable Cox regressions are used to calculate the hazard ratios (HR) with 95% confidence interval (CI).

CDR (day*10^{-3}/L)	All-cause graft failure		Death-censored graft failure		Patient mortality	
	**HR**	**95% CI** ***P* value**	**HR**	**95% CI** ***P* value**	**HR**	**95% CI** ***P* value**
<0.58	**1.93**	1.61–2.32<br><0.001	**3.11**	2.47–3.92<br><0.001	1.11	0.82–1.49<br>0.51
0.58–<0.78	**1.56**	1.30–1.87<br><0.001	**1.99**	1.55–2.56<br><0.001	1.25	0.96–1.62<br>0.092
0.78–<1.05	**1.26**	1.07–1.48<br>0.006	**1.57**	1.25–1.97<br><0.001	0.99	0.79–1.25<br>0.95
1.05–<1.42	0.98	0.84–1.15<br>0.81	1.20	0.96–1.50<br>0.11	0.83	0.67–1.03<br>0.096
≥1.42	1.00	Ref.	1.00	Ref.	1.00	Ref.

Table S4. Influence of 1-year trough level/dose ratio (CDR, day*10^{-3}/L) on death-censored graft failure during second and third post-transplant years in different subpopulations. Univariable Cox regressions were used to calculate the hazard ratios (HR) with 95% confidence interval (CI) and CDR ≥1.05 as reference.

**Subpopulation**	**N**	**CDR<0.58**			**CDR 0.58 – <1.05**		
		**HR**	**95 % CI**	***P***	**HR**	**95 % CI**	***P***
All patients	21,865	**2.98**	2.38–3.73	<0.001	**1.65**	1.38–1.97	<0.001
Recipient sex							
Female	8,440	**3.80**	2.79–5.18	<0.001	**1.55**	1.18–2.04	0.002
Male	13,425	**2.29**	1.64–3.20	<0.001	**1.73**	1.37–2.18	<0.001
Recipient age							
18–49 y	11,849	**3.25**	2.50–4.22	<0.001	**1.67**	1.34–2.09	<0.001
50–59 y	5,421	**2.57**	1.44–4.61	0.001	**1.95**	1.32–2.88	<0.001
≥60 y	4,595	1.51	0.66–3.44	0.33	1.07	0.64–1.79	0.80
Donor relationship							
Deceased	15,446	**3.20**	2.50–4.09	<0.001	**1.76**	1.45–2.13	<0.001
Living	6,419	**2.47**	1.42–4.32	0.001	1.25	0.78–2.00	0.36
1-year serum creatinine							
<130 µmol/L	12,829	**2.81**	1.68–4.72	<0.001	**1.88**	1.30–2.73	<0.001
≥130 µmol/L	9,036	**2.56**	2.00–3.29	<0.001	**1.42**	1.16–1.74	<0.001
1-year trough level							
<4.5 ng/mL	1,888	**2.06**	1.33–3.18	0.001	1.18	0.75–1.86	0.47
≥4.5 ng/mL	19,977	**2.68**	2.02–3.55	<0.001	**1.64**	1.35–1.99	<0.001
1-year steroid dose							
≤5.0 mg/day	17,385	**2.96**	2.26–3.89	<0.001	**1.79**	1.46–2.20	<0.001
>5.0 mg/day	3,427	**2.53**	1.65–3.86	<0.001	1.31	0.90–1.92	0.16

Table S5. Odds ratios (OR) with 95% confidence interval of variables with significant influence on a low 1-year CDR below 1.05 day*10^{-3}/L in univariable and multivariable logistic regression analysis.

Covariate	Univariable			Multivariable		
	**OR**	**95% CI**	***P* value**	**OR**	**95% CI**	***P* value**
Overall study population (n=21,865)						
Black recipient	**2.43**	2.20–2.68	<0.001	**2.32**	2.09–2.56	<0.001
Age 50–59 vs. <50	**0.47**	0.43–0.51	<0.001	**0.51**	0.47–0.56	<0.001
Age ≥60 vs. <50	**0.63**	0.58–0.68	<0.001	**0.65**	0.60–0.70	<0.001
Female recipient	**1.42**	1.33–1.51	<0.001	**1.39**	1.30–1.48	<0.001
Diabetic recipient	**0.68**	0.62–0.74	<0.001	**0.75**	0.69–0.82	<0.001
Genotyped subpopulation (n=1,257)						
CYP3A5 with *1A	**8.19**	4.96–13.5	<0.001	**8.10**	4.84–13.6	<0.001
CYP3A4 with *22	0.41	0.13–1.32	0.14	0.62	0.19–2.08	0.44
Black recipient*	–	–	–	–	–	–
Age 50–59 vs. <50	0.70	0.40–1.25	0.23	0.66	0.36–1.20	0.17
Age ≥60 vs. <50	0.57	0.31–1.04	0.068	0.68	0.35–1.30	0.24
Female recipient	**1.64**	1.01–2.66	0.045	1.52	0.91–2.55	0.11
Diabetic recipient	0.80	0.41–1.55	0.51	0.87	0.43–1.77	0.71

* no black recipients

Figure S1. Comparison of overall graft survival of patients with known *CYP3A4/CYP3A5* genotyping compared to that of patients without typing (only patients of European ancestry without the region Latin America).

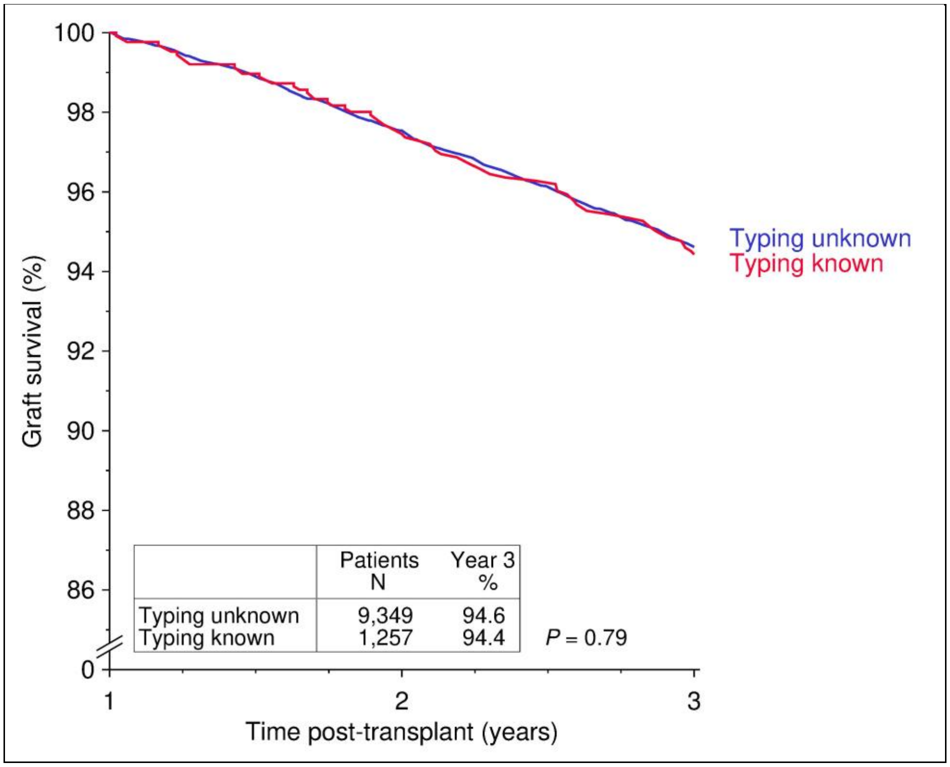

Figure S2. Correlation of diltiazem use at year 1 with 1-year tacrolimus trough level/dose ratio (CDR).

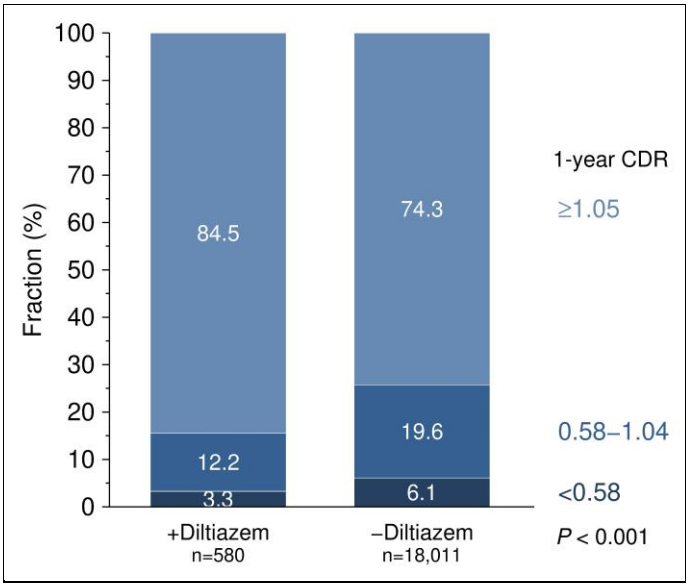

Figure S3. Correlation of steroid dosage at year 1 (low dose: ≤5 mg/day; high dose: >5 mg/day) with 1-year tacrolimus trough level/dose ratio (CDR).

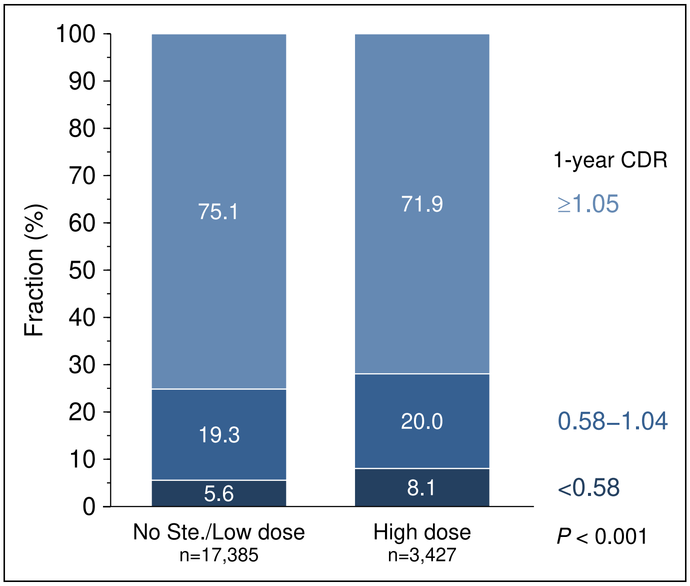

Supplement: Supplementary file 1 [file DataSheet1.pdf]
